# Perception Towards Epidemiology of Human Immunodeficiency Virus or Acquired Immunodeficiency Syndrome Among Women of Reproductive Age: Analysis of Nationwide Surveys in India

**DOI:** 10.7759/cureus.41643

**Published:** 2023-07-10

**Authors:** Ranganath TS, Era Gupta, Shoyaib K Md

**Affiliations:** 1 Community Medicine, Bangalore Medical College and Research Institute, Bengaluru, IND

**Keywords:** attitude, knowledge, demographic and health survey, hiv, women

## Abstract

Introduction: The knowledge of HIV prevention, attitudes towards people living with HIV (PLHIV), and beliefs in myths related to HIV/AIDS among women of reproductive age group in India hold immense importance. As this group is particularly vulnerable to HIV infection, having accurate knowledge about prevention methods is crucial to protect themselves and their partners. Positive attitudes towards PLHIV foster support, reduce stigma and encourage early testing and treatment. Additionally, debunking myths and misconceptions surrounding HIV/AIDS can help dispel fear and promote informed decision-making. Empowering women with comprehensive knowledge, fostering positive attitudes, and debunking myths can contribute to effective HIV prevention strategies, reduce transmission rates, and improve women's overall well-being in India.

Objectives: Our objective was to create a regression model to show disparities in knowledge of the prevention of HIV/AIDS, attitude towards people living with HIV/AIDS, and belief in myths among women in India with adjusted odds ratio (aOR) for different socio-demographic factors, and to determine if there are any significant changes in the aOR during both surveys.

Study Design: A comparative study using secondary data analysis of two complex sample surveys.

Methods: Individual Recode files from datasets obtained from Demographic Health Surveys (DHS) Program were exported to STATA v17.0 MP by STATA Corp LLC. Dependent and independent variables were chosen based on a literature review and computed. The design-adjusted chi-square test was used to assess the association between variables. A multinomial logistic regression analysis was used to derive a model. The model assessment was performed using the Wald test after estimation.

Results: The age group of 15-19 years had the highest odds of having sufficient knowledge of the prevention of HIV/AIDS during both National Family Health Surveys (NFHS) (aOR: 1.98 during NFHS-4 and aOR: 1.80 during NFHS-5). No education group had the highest odds of negative attitudes towards people living with HIV/AIDS during both surveys (aOR: 4.85 during NFHS-4 and aOR: 2.69 during NFHS-5). Rural areas had higher odds of believing in myths related to HIV/AIDS during both surveys (aOR: 1.07 during NFHS-4 and aOR: 1.13 during NFHS-5).

Conclusions: This research study sheds light on the significant relationship between demographic factors and knowledge, attitudes, and beliefs related to HIV/AIDS among women. Specifically, women in the lower age group, lacking education, and residing in rural areas exhibited substantial levels of inadequate knowledge regarding HIV/AIDS prevention. Furthermore, this group was also associated with higher proportions of negative attitudes towards people living with HIV/AIDS and a greater tendency to believe in myths associated with HIV/AIDS.

## Introduction

HIV was first detected in India in 1986 [1]. According to the National AIDS Control Organization (NACO), HIV prevalence among adults in India is approximately 0.22%, with women accounting for a considerable proportion of cases [2]. According to the India HIV Estimations 2017 report, an estimated 40% of new HIV infections in India occurred among women aged 15-45 years [3]. Despite the improvement in knowledge, women consistently lag behind men, reflecting the broader context of gender relations in India, which limits women's access to education in general and health-related knowledge in particular [4]. Fear of unknown diseases and guilt leads to stigma, discrimination, and victimization [5].

Knowledge and attitudes are essential determinants in changing health behaviors, as is well documented in various health behavior theories. According to a study conducted among adults in Kolkata by Porter et al. [6], more than one-third of the respondents would not eat or continue to work with AIDS patients, and more than half believed that HIV/AIDS patients should be quarantined. Mass media campaigns are associated with an increased awareness of HIV/AIDS among people in developing countries. Knowledge focuses on the epidemiology of HIV/AIDS. Media also plays an essential role in constructing and changing beliefs and attitudes, two factors critical to health behaviors. While there has been a good increase in the number of women using television and print media as a source of information, this was not the case among underprivileged women [7]. This requires alternate modes of information dissemination related to HIV/AIDS.

Moreover, Pallikadavath S et al., from their study recommended new innovative strategies to disseminate knowledge among unprivileged groups [4]. Also, knowledge of HIV/AIDS and adolescent sexual behavior is fascinating because, for many young people, the period between sexual initiation and marriage is a time of sexual experimentation that may involve risky behaviors. Narrowing the gender gap in new infections is an equally important concern, suggesting that the problems and concerns of young people need to be addressed urgently [5]. In addition, Ackerson et al. [8] concluded that knowledge is associated with reduced stigma toward people living with HIV/AIDS. 

Stigma, on the other hand, is a recognized obstacle to the early detection of HIV and causes great suffering for those affected [9]. One such example can be seen in the research by Ananth et al., which was carried out in cities such as Delhi, Mumbai, Bangalore, and Kollam among adult women, where more than half of the respondents advocated for abortion among seropositive pregnant women [10]. Moreover, female respondents in a survey were found to be more resistant to change due to various cultural and social norms [5]. Knowledge and beliefs about HIV/AIDS in the population will likely affect the treatment of people with HIV/AIDS. Adolescents who are relatively better informed about HIV/AIDS than their elders are expected to be more accepting of people living with HIV/AIDS [11].

India was projected to surpass China as the world's most populous country in 2023 [12]. Globally around 1.5 million people became newly infected with HIV in 2021, and every week, around 4900 young women aged 15-24 years become infected with HIV [13]. For such a disease affecting people across the globe, increased awareness of HIV among women in India can have significant global implications for reducing transmission rates, improving health outcomes, increasing access to prevention methods, reducing stigma, and fostering greater global cooperation.

This study assesses the influence of socio-demographic factors such as age, education, region of residence, type of area of residence, and exposure to mass media on knowledge of the prevention of HIV/AIDS, attitude towards people living with HIV/AIDS, and beliefs in myths related to HIV/AIDS. Our objective was to derive a model to show the disparities in knowledge about HIV/AIDS prevention, attitudes towards people living with HIV/AIDS, and belief in myths among women across the country and assess how different socio-demographic factors impact different aspects of perception.

## Materials and methods

Study design

A comparative study using secondary data analysis of complex sample surveys of a nationally representative population.

Setting

NFHS 4 was carried out in six hundred forty districts in twenty-nine states and six union territories, and NFHS 5 was carried out in seven hundred and seven districts in twenty-eight states and eight union territories.

Sample characteristics

**Figure 1:**
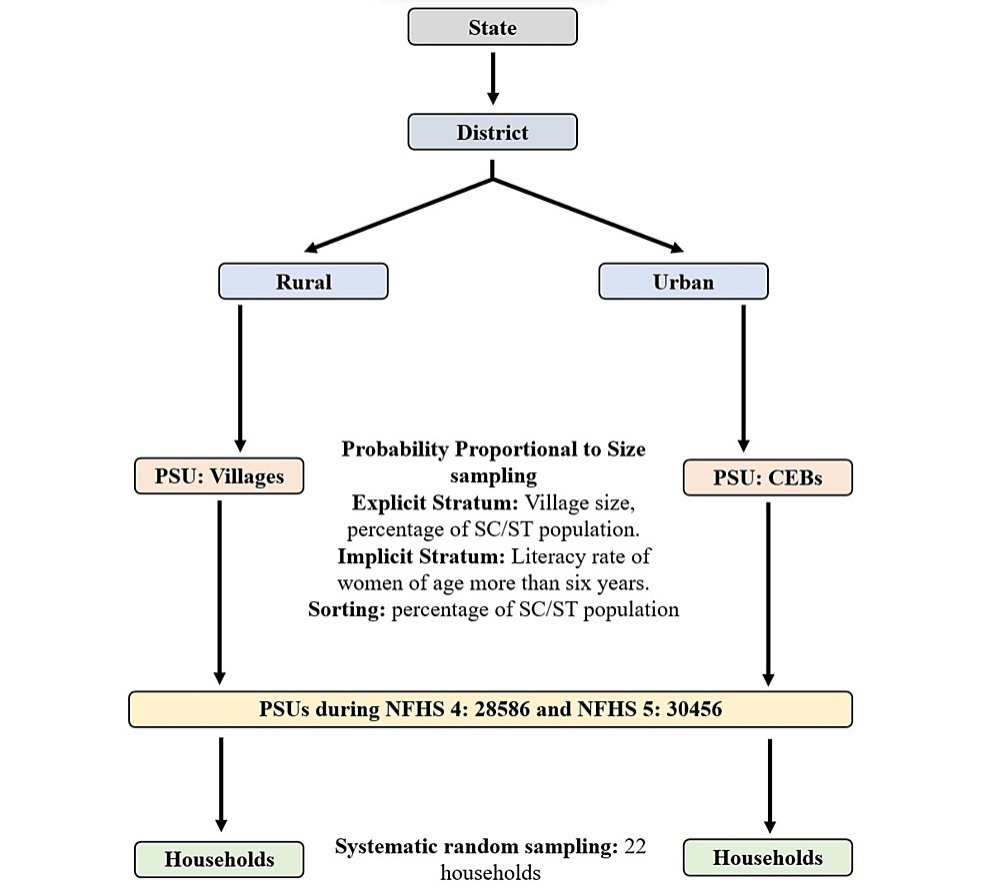
Flowchart showing sampling for households during NFHS 4 and NFHS 5. PSU: Primary Sampling Unit; CEB: Census Enumeration Block; NFHS: National Family Health Survey

The NFHS-4 and NFHS-5 samples are stratified two-stage. The 2011 census was the sampling frame for selecting Primary Sampling Units (PSUs). PSUs were villages in rural areas and Census Enumeration Blocks (CEBs) in urban areas. PSUs with fewer than forty households were linked to the nearest PSU. Within each rural stratum, villages were selected from the sampling frame with probability proportional to size (PPS). In each stratum, six approximately equal substrata were created by crossing three substrata, each created based on the estimated number of households in each village, with two substrata, each created based on the percentage of the population belonging to scheduled castes and scheduled tribes (SCs/STs). Within each explicit sampling stratum, PSUs were sorted according to the literacy rate of women aged above six years. The final sample PSUs were selected with PPS sampling. In urban areas, CEB information was obtained from the Office of the Registrar General and Census Commissioner, New Delhi. CEBs were sorted according to the SC/ST population percentage in each CEB, and sample CEBs were selected with PPS sampling. A complete household mapping and listing operation was conducted before the primary survey in every selected rural and urban PSU. Selected PSUs with an estimated number of at least three hundred households were segmented into segments of approximately one hundred to one hundred fifty households. Two segments were randomly selected for the survey using systematic sampling with probability proportional to segment size. Therefore, an NFHS-4 cluster is either a PSU or a segment of a PSU. In the second stage, twenty-two households were randomly selected with systematic sampling in every selected rural and urban cluster. The flow chart is given in Figure 1 [14,15].

Participants

**Figure 2:**
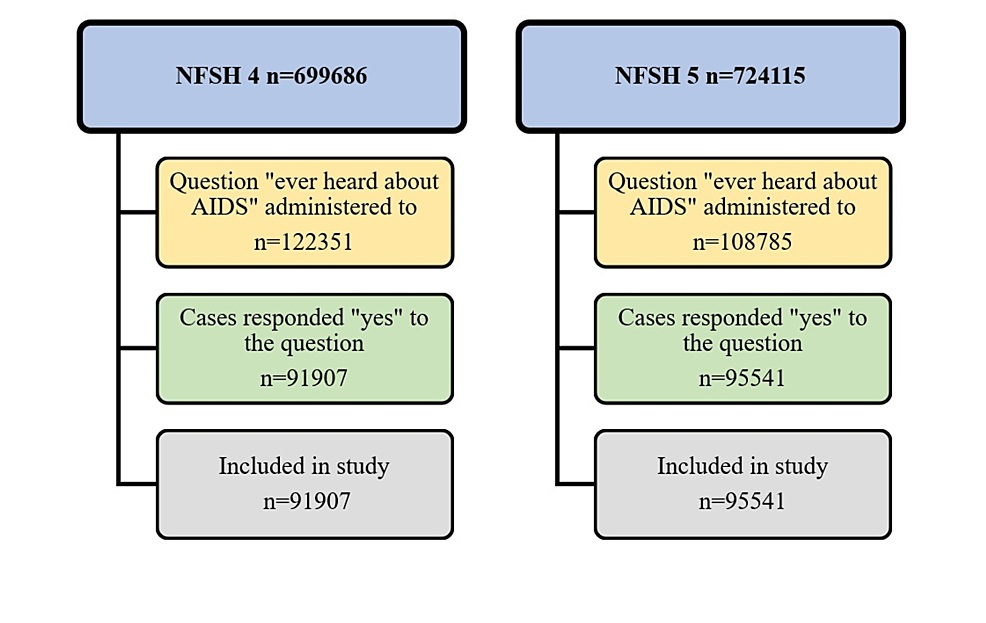
Selection of cases from Individual Recode file of NFHS 4 and NFHS 5 for analysis

Both surveys interviewed women in the age group of 15-45 years. In NFHS 4 dataset, 699686 cases were found in the Individual Recode (IR) file. It contains details of all the women interviewed during NFHS 4. Similarly, 724115 cases were found in the IR file of the NFHS 5 dataset. Our inclusion criteria to include in our analysis were all those women who answered "yes" to the question "Ever heard about AIDS". The total number of cases included for analysis from both surveys is given in Figure 2. All the women to whom the question "Ever heard about AIDS" was not administered or those who answered "no" to the same question were excluded from the analysis.

Dependent variables

For each question included in the calculation of the dependent variables, the Demographic and Health Surveys (DHS) assigned a score of "0" for a "no" answer, a score of "1" for a "yes" answer, and a score of "8" for a "do not know" answer.

Knowledge of HIV Prevention

Calculated by adding the scores of two variables. "Reduce HIV risk: Always use condoms during sex" and "Reduce HIV risk: only have one sexual partner who has no other partners." A final score of "2" after the addition was considered sufficient knowledge about HIV prevention and the reference category for the analysis.

Attitude Toward People Living with HIV

Calculated by summing up three variables: "A healthy-looking person may have HIV", "willing to care for a relative with AIDS", and "An HIV-infected but not sick teacher should be allowed to continue teaching". A final score of "3" after the addition was considered positive attitudes toward people living with HIV and the reference category for the analysis.

Beliefs in Myths Related to HIV

Calculated by adding scores from two variables. "Can get HIV from sharing food with a person who has AIDS" and "Can get HIV from mosquito bites". A final score of "0" after the addition was considered "No belief in HIV-related myths" and was considered the reference category for the analysis.

Data Sources

Datasets from DHS, also known as National Family Health Survey (NFHS) in India. We analyzed the data from the Individual recode file, and Men recode file of NFHS 4 and NFHS 5 datasets.

Independent variables

The current age in 5-year groups. The age group of 45-49 years was taken as the reference category.

Highest Educational Level

This standardized variable provides the level of education in the following categories: No education, Primary, Secondary, and Higher [16]. The higher education group was taken as the reference category.

Type of Place of Residence

Residence in a rural or urban type of area [16]. Urban type of place of residence was taken as the reference category. Zones: All the states and union territories were combined into zones as per zonal councils according to the States Reorganization Act of 1956 [17]. The South zone was taken as the reference category.

Exposure to Mass Media

It was calculated by adding up three variables: "frequency of reading a newspaper", "frequency of listening to the radio," and "frequency of television viewing" [16]. For all three variables, there were four possible options with scoring, "0" for not at all, "1" for less than once a week, "2" for at least once a week, and "3" for almost every day. The highest value was taken as the reference category.

Study Size

Based upon the fulfillment of inclusion criteria, 91907 cases from NFHS 4 and 95541 cases from NFSH 5 were included in the analysis. 

Statistical Methods

Appropriate adjustments were made before starting the analysis, such as the declaration of complex survey design by defining the variables for PSUs, Strata, and sampling weights. The commands below were used under the subset of the "svy" command in STATA MP v17 by Stata Corp LLC. A design-adapted chi-square test was used to assess for an association between categorical variables. Next, we created a multinomial logistic regression model. Finally, a multiparametric Wald test was performed for all variables added to the regression model.

## Results

A total of 91907 cases from NFHS 4 and 95541 cases from NFHS 5 were included in our final analysis of the regression model to derive an adjusted odds ratio (aOR).

Descriptive Data

In NHFS 4, the distribution of respondents by 5-year age group was nearly uniform across all groups, with 18.38% being in the 20-24 age group, followed by the 25-29 age group with 17.02%. The lowest number of respondents was found in the 45-49 age group, 9.92%. Similarly, in the NFHS 5, the respondents in the 20-24 age group were 16.77%, followed by 25-29 years old at 16.52%. At 10.98%, the most minor proportion of participants was in the 40 to 44 age group. For the level of education, in NFHS 4, 53.84% of respondents had a secondary education, followed by 17.82% with no education. Only 17.40% had higher education. A similar proportion pattern was found among respondents during NFHS 5, where 51.64% had secondary education, followed by 19.62% of respondents with no education. However, a minor percentage was among respondents with primary education, 11.03%. For the distribution of respondents in zones, in NFHS 4, most respondents were from the South Zone at 30.20%, followed by the East Zone, i.e., 17.71%. The smallest proportion of respondents came from the Northeast Zone, which was 03.55%. Similarly, during the NFHS 5 survey, 22.58% of respondents were from the South Zone, followed by 21.66% from the East Zone. The smallest proportion of respondents were from the Northeast Zone, i.e., 04.01%. As per the type of area of residency, 41.46% of respondents lived in urban areas, and the rest were in rural areas during NFHS 4. However, the proportion of residents in urban areas was only 34.47%, and the rest lived in urban areas during NFHS 5. Most respondents, i.e., 71.71% and 72.08%, were married during NFHS 4 and NFHS 5, respectively. More details are given in (Table 1).

**Table 1:** Descriptive univariate analysis of socio-demographic details of the women of reproductive age group as per NFHS 4 and NFHS 5 included in the analysis. NFHS: National Family Health Survey

	NFHS 4 (2015-16)	NFHS 5 (2019-21)
	Number of cases for analysis: n = 91907	Number of cases for analysis: n = 95541
Age Group in years		
15-19	16.78%	15.96%
20-24	18.38%	16.77%
25-29	17.02%	16.52%
30-34	14.37%	14.50%
35-39	12.81%	13.64%
40-44	10.72%	10.98%
45-49	9.92%	11.63%
Education		
No education	17.82%	19.62%
Primary	10.94%	11.04%
Secondary	53.84%	51.64%
Higher	17.40%	17.70%
Zones		
North	13.69%	14.04%
Northeast	03.55%	04.01%
Central	18.72%	24.12%
East	17.71%	21.66%
West	16.13%	13.59%
South	30.20%	22.58%
Type of area of Residence		
Urban	41.46%	34.47%
Rural	58.36%	65.53%
Religion		
Hindu	79.70%	18.97%
Non-Hindu	20.30%	81.03%
Current Marital Status		
Never Married	24.15%	23.58%
Married	71.71%	72.08%
Others	04.14%	04.34%

The distribution of prevalence of insufficient knowledge about the prevention of HIV/AIDS, negative attitude towards People living with HIV (PLHIV), and beliefs in myths related to HIV/AIDS across various factors during NFHS 4 and NFHS 5 are given in (Table 2).

**Table 2:** Prevalence of insufficient knowledge of prevention of HIV/AIDS, negative attitude towards PLHIV, and beliefs in myths related to HIV/AIDS across various socio-demographic factors during NFHS 4 and NFHS 5. HIV: Human Immunodeficiency Virus; AIDS: Acquired Immunodeficiency Syndrome; PLHIV: People living with HIV

	Prevalence of insufficient Knowledge of prevention of HIV/AIDS		Prevalence of Negative attitude towards PLHIV		Prevalence of beliefs in myths related to HIV/AIDS	
	NFHS 4	NFHS 5	NFHS 4	NFHS 5	NFHS 4	NFHS 5
Age Group in years						
15-19	2.95%	2.31%	1.01%	1.06%	2.23%	3.19%
20-24	2.26%	1.49%	1.05%	0.90%	2.10%	3.18%
25-29	2.00%	1.31%	1.12%	0.93%	1.97%	3.03%
30-34	1.74%	1.21%	0.98%	0.80%	1.72%	2.47%
35-39	1.64%	1.19%	0.95%	0.83%	1.67%	2.50%
40-44	1.68%	1.07%	0.90%	0.63%	1.45%	2.09%
45-49	1.52%	1.34%	0.90%	0.83%	1.49%	2.34%
Education						
No education	3.71%	2.64%	2.09%	1.68%	3.30%	5.06%
Primary	1.92%	1.28%	0.94%	0.86%	1.66%	2.37%
Secondary	6.98%	5.08%	3.39%	2.88%	6.34%	9.24%
Higher	1.18%	0.93%	0.48%	0.57%	1.33%	2.14%
Zones						
North	1.24%	1.15%	0.55%	0.65%	1.86%	2.52%
Northeast	1.00%	0.65%	0.31%	0.27%	0.48%	0.96%
Central	2.05%	2.37%	0.98%	1.78%	2.76%	5.69%
East	2.88%	2.55%	1.08%	1.22%	2.54%	4.82%
West	1.78%	1.04%	0.87%	0.51%	1.40%	1.42%
South	4.84%	2.17%	3.12%	1.56%	3.59%	3.41%
Type of area of Residence						
Urban	4.67%	2.69%	2.49%	1.61%	4.15%	5.10%
Rural	9.12%	7.23%	4.42%	4.38%	8.48%	13.71%

Outcome Data

Adjusted odds ratio for various factors involved in the regression model for no knowledge of prevention of HIV/AIDS, negative attitude towards PLHIV, and believing in myths related to HIV/AIDS.

Main results

The adjusted odds ratio for knowledge of prevention of HIV/AIDS: The respondents in the age group of 15-19 years had the highest likelihood of having insufficient knowledge about HIV/AIDS prevention in both surveys (aOR: 1.98 during NFHS-4 and aOR: 1.80 during NFHS-5). Moreover, no education was associated with a maximum likelihood of insufficient knowledge of HIV/AIDS prevention among all education groups at both assessments (aOR: 3.66 during NFHS-4 and 2.45 during NFHS-5). In addition, respondents residing in the Northeast zone had the highest odds of poor knowledge of HIV/AIDS prevention in both surveys (aOR: 1.77 during NFHS-4 and 1.46 during NFHS-5). Also, in both surveys, respondents living in rural areas were likelier to have insufficient knowledge about HIV/AIDS prevention (aOR: 1.15 during NFHS-4 and 1.14 during NFHS-5). However, A higher frequency of mass media exposure was associated with a lower likelihood of having insufficient knowledge about HIV/AIDS prevention in both surveys (aOR: 0.90 during NFHS-4 and NFHS-5). More details are given in (Table 3).

**Table 3:** Multinomial Regression model showing adjusted odds ratio for inadequate knowledge of prevention of HIV among women of reproductive age group during NFHS 4 and NFHS 5 survey. p-value <0.05 for *Wald Test p-value <0.05 for **adjusted Odds Ratio ^ Confidence Interval for adjusted Odds Ratio for NFHS 4 and NFHS 5 does not overlap NFHS: National Family Health Survey

Knowledge of prevention of HIV (Reference category : Sufficient)	NFHS 4 (2015-16)	NFHS 5 (2019-21)
	Number of cases for analysis: 91907	Number of cases for analysis: 95541
	Design df = 7,269 F(32, 7238) = 54.85 Prob > F <0.001	Design df = 6,403 F(32, 6372) = 35.94 Prob > F <0.001
Age Group in years	F_12, 7258_=26.80 P(F>F) < 0.001*	F_12, 6392_=28.07 P(F>F) < 0.001*
15-19	1.98 (1.76 - 2.22)**	1.80 (1.59 - 2.03)**
20-24	1.23 (1.09 - 1.40)**	1.05 (0.93 - 1.19)
25-29	0.98 (0.87 - 1.11)	0.84 (0.74 - 0.95)**
30-34	0.96 (0.85 - 1.09)	0.81 (0.71 - 0.91)**
35-39	0.90 (0.80 - 1.03)	0.80 (0.71 - 0.90)**
40-44	1.11 (0.98 - 1.25)	0.86 (0.76 - 0.98)**
45-49	Reference Category	
Education	F_6, 7264_=72.09 P(F>F) < 0.001*	F_4, 6400_=36.95 P(F>F) < 0.001*
No education	3.66 (3.18 - 4.20)**	2.45 (2.08 - 2.88)**^
Primary	2.89 (2.49 - 3.34)**	2.10 (1.79 - 2.46)**^
Secondary	1.74 (1.56 - 1.95)**	1.59 (1.38 - 1.83)**
Higher	Reference Category	
Zone	F_10, 7260_=87.42 P(F>F) < 0.001*	F_10, 6394_=21.95 P(F>F) < 0.001*
North	0.36 (0.31 - 0.41)**	0.65 (0.57 - 0.75)**^
Northeast	1.77 (1.55 - 2.03)**	1.46 (1.26 - 1.70)**
East	0.41 (0.37 - 0.46)**	0.75 (0.65 - 0.85)**^
Central	0.67 (0.58 - 0.77)**	0.93 (0.81 - 1.08)
West	0.55 (0.46 - 0.65)**	0.71 (0.58 - 0.87)**
South	Reference Category	
Type of area of Residence	F_2, 7268_=4.17 P(F>F)= 0.02*	F_2, 6402_=3.70 P(F>F) < 0.001*
Rural	1.15 (1.04 - 1.27)**	1.14 (1.01 – 1.28)***
Urban	Reference Category	
Exposure to Mass Media	F_2, 7268_=51.42 P(F>F) < 0.001*	F_2, 6402_=3.78 P(F>F)= 0.02*
	0.90 (0.88 - 0.92)**	0.85 (0.83 – 0.88)**

The adjusted odds ratio for attitude towards PLHIV: Respondents aged 15-19 years had the highest likelihood of having negative attitudes toward PLHIV in both surveys (aOR: 1.02 during NFHS-4 and aOR: 1.26 during NFHS-5). Similar to respondents in the Adjusted odds ratio for knowledge of prevention of HIV/AIDS, the same group, that is, those with no education, had the highest likelihood of having negative attitudes towards PLHIV in both surveys (aOR: 4.85 during NFHS-4 and aOR: 2.69 during NFHS-5). However, regarding negative attitudes toward PLHIV, the Northeast Zone had the lowest likelihood of negative attitudes (aOR: 0.55) during the NFHS-4 survey. In contrast, the East Zone had the lowest likelihood of negative attitudes (aOR: 0.74). Discordant to the above, in both surveys, the rural area was associated with a higher likelihood of negative attitudes toward PLHIV (aOR: 1.05 during NFHS-4 and aOR: 1.18 during NFHS-5). At the same time, a higher frequency of exposure to mass media was associated with a lower likelihood of negative attitudes toward PLHIV in both surveys. However, it was not statistically significant during the NFHS-4 survey (aOR: 0.84 during NFHS-4 and aOR: 0.82 during NFHS-5). More details are given in (Table 4).

**Table 4:** Multinomial Regression model showing adjusted odds ratio for negative attitude towards people living with HIV among women of reproductive age group during NFHS 4 and NFHS 5 survey. p-value <0.05 for *Wald Test p-value <0.05 for ** adjusted Odds Ratio ^ Confidence Interval for adjusted Odds Ratio for NFHS 4 and NFHS 5 does not overlap NFHS: National Family Health Survey

Attitude towards people living with HIV (Reference category: Positive)	NFHS 4 (2015-16)	NFHS 5 (2019-21)
	Number of cases for analysis: 91907	Number of cases for analysis: 95541
	Design df = 7,269 F(32, 7238) = 54.85 Prob > F <0.001	Design df = 6,403 F(48, 6356) = 25.95 Prob > F <0.001
Age Group in years	F_36_, _7234_=2.03 P(F>F) < 0.001*	F_18, 6386_=3.74 P(F>F) < 0.001*
15-19	1.02 (0.83 - 1.24)	1.26 (1.06 - 1.48)**
20-24	0.98 (0.80 - 1.21)	1.08 (0.92 - 1.28)
25-29	0.97 (0.82 - 1.15)	1.03 (0.89 - 1.19)
30-34	0.88 (0.72 - 1.08)	0.88 (0.75 - 1.04)
35-39	0.86 (0.71 - 1.05)	0.92 (0.79 - 1.08)
40-44	0.98 (0.80 - 1.21)	0.83 (0.71 - 0.98)**
45-49	Reference Category	
Education	F_12_, _7258_=29.62 P(F>F) < 0.001*	F_6, 6398_=35.95 P(F>F) < 0.001*
No education	4.85 (3.84 - 6.13)**	2.69 (2.24 - 3.24)**^
Primary	3.49 (2.75 - 4.44)**	2.63 (2.19 - 3.16)**
Secondary	2.47 (2.06 - 2.96)**	1.79 (1.54 - 2.09)**
Higher	Reference Category	
Region	F_30, 7240_=30.59 P(F>F) < 0.001*	F_15, 6389_=38.93 P(F>F) < 0.001*
North	0.23 (0.19 - 0.27)**	0.42 (0.35 - 0.49)**^
Northeast	0.55 (0.45 - 0.66)**	0.46 (0.38 - 0.56)**
East	0.29 (0.25 - 0.34)**	0.74 (0.63 - 0.87)**^
Central	0.32 (0.27 - 0.38)**	0.46 (0.38 - 0.56)**
West	0.41 (0.32 - 0.51)**	0.36 (0.29 - 0.45)**
South	Reference Category	
Type of area of Residence	F_6, 7264_=2.43 P(F>F)= 0.02*	F_3, 6401_=3.70 P(F>F)= 0.01*
Rural	1.06 (0.91 - 1.22)	1.19 (1.04 – 1.35)**
Urban	Reference Category	
Exposure to Mass Media	F_6, 7264_=39.45 P(F>F) < 0.001*	F_2, 6401_=41.18 P(F>F) < 0.001*
	0.84 (0.82 - 0.87)	0.83 (0.78 – 0.87)**

The adjusted odds ratio for beliefs in myths related to HIV/AIDS: Respondents with no education had the highest odds of believing HIV/AIDS-related myths (aOR: 1.65 during NFHS-4 and aOR: 1.53 during NFHS-5), and those belonging to the Central Zone had the highest odds (aOR: 1.40) during the NFHS-4 compared to the East Zone (aOR: 1.71) during the NFHS-5. Similar to the previous two aspects, respondents in rural areas were more likely to believe HIV/AIDS-related myths in both surveys (aOR: 1.07 during NFHS-4 and aOR: 1.13 during NFHS-5). Moreover, in both surveys, a higher frequency of exposure to mass media was also associated with lower odds of believing HIV/AIDS-related myths (aOR: 0.97 during NFHS-4 and NFHS-5). More details are given in (Table 5).

**Table 5:** Multinomial Regression model showing adjusted odds ratio for beliefs in myths related to HIV among women of reproductive age group during NFHS 4 and NFHS 5 survey. p-value <0.05 for *Wald Test p-value <0.05 for ** adjusted Odds Ratio ^ Confidence Interval for adjusted Odds Ratio for NFHS 4 and NFHS 5 does not overlap NFHS: National Family Health Survey

Beliefs in myths related to HIV (Reference category: No)	NFHS 4 (2015-16)	NFHS 5 (2019-21)
	Number of cases for analysis: 91907	Number of cases for analysis: 95541
	Design df = 7,269 F(32, 7238) = 20.89 Prob > F <0.001	Design df = 6,403 F(32, 6372) = 39.83 Prob > F <0.001
Age Group in years	F_12, 7258_=2.61 P(F>F)= 0.01*	F_12, 6392_=3.74 P(F>F) < 0.001*
15-19	1.03 (0.94 - 1.14)	1.09 (0.99 - 1.18)
20-24	1.05 (0.95 - 1.15)	1.06 (0.97 - 1.14)
25-29	0.98 (0.89 - 1.08)	0.93 (0.85 - 1.01)
30-34	0.95 (0.86 - 1.05)	0.90 (0.83 - 0.99)**
35-39	0.95 (0.87 - 1.05)	0.95 (0.87 - 1.03)
40-44	0.98 (0.89 - 1.08)	0.94 (0.86 - 1.03)
45-49	Reference Category	
Education	F_6, 7264_=3630 P(F>F) < 0.001*	F_4, 6400_=36.68 P(F>F) < 0.001*
No education	1.65 (1.49 - 1.83)**	1.54 (1.41 - 1.68)**
Primary	1.51 (1.37 - 1.67)**	1.40 (1.29 - 1.54)**
Secondary	1.36 (1.27 - 1.47)**	1.23 (1.15 - 1.32)**
Higher	Reference Category	
Zone	F_10, 7260_=16.47 P(F>F) < 0.001*	F_10, 6394_=47.73 P(F>F) < 0.001*
North	1.23 (1.12 - 1.34)**	1.267 (1.16 - 1.37)**
Northeast	0.99 (0.90 - 1.10)	1.10 (0.99 - 1.21)
East	1.39 (1.292 - 1.510)**	1.71 (1.58 - 1.86)**^
Central	1.40 (1.272 - 1.551)**	1.45 (1.32 - 1.59)**
West	0.96 (0.87 - 1.06)	0.77 (0.70 - 1.59)**
South	Reference Category	
Type of area of Residence	F_2, 7268_=12.52 P(F>F) < 0.001*	F_2, 6402_=22.88 P(F>F) < 0.001*
Rural	1.07 (1.01 - 1.14)**	1.13 (1.06 - 1.21)*
Urban	Reference Category	
Exposure to Mass Media	F_2, 7268_=14.37 P(F>F) < 0.001*	F_2, 6402_=11.65 P(F>F) < 0.001*
	0.97 (0.95 - 0.98)**	0.97 (0.95 – 0.98)**

## Discussion

The highest odds of having insufficient knowledge of the prevention of HIV/AIDS were seen among women of younger age groups, those with no education, and those residing in the Northeast zone and Rural areas. Similar findings were seen regarding higher odds of having a negative attitude towards PLHIV, except for region, in which the highest odds were among women residing in the Northeast zone and East zone during NFHS 4 and NFHS 5, respectively. With respect to odds of believing in myths related to HIV/AIDS, a similar pattern of groups had the highest odds as that for knowledge regarding the prevention of HIV/AIDS except for a region in which, during NFHS 4, the Central zone had the highest odds and during NFHS 5 East zone had the highest odds.

In 2019 at the national level, an estimated 23.49 lakh people were living with HIV, of which 3.4% were children, and women above the age of 15 constituted around 44% [2]. Although HIV prevention activities initially focused on high-risk groups such as intravenous drug users, female sex workers, and male truck drivers, the epidemic is more generalized, with particular burdens faced by residents with fewer privileges such as low education, low socioeconomic status, etc. [8]. Moreover, antenatal mothers have less awareness and wrong perceptions [18], a risk factor for vertical transmission of HIV.

In this research, only the youngest age group of women, i.e., 15 to 19 years, were associated with higher odds of insufficient knowledge of the prevention of HIV during NFHS 4 and NFHS 5. This can be attributed to the fact that sex education is still taboo in most parts of the country, and there is an intense requirement for the same [19]. However, the increase in age was associated with lesser odds of insufficient knowledge during NFHS 5 only, which can be attributed to available sources of information such as smartphones with cheaper and faster internet access than before. All levels of education were the risk factor for insufficient knowledge on the prevention of HIV, which reflects that there was not much information available in the literature on formal education regarding HIV prevention. Rural women are more underprivileged due to stricter customs and gender norms and are associated with higher odds of insufficient information. Similarly, [2,3,14] report confirmed that proper knowledge is still low, particularly in the rural area.

Moreover, another study published in 2011 [8] showed that knowledge about preventing and transmitting AIDS was negatively associated with HIV/AIDS-related stigma. Mass media may help reduce social disparities in HIV/AIDS outcomes. According to a study done in 2006 [7], television was the most effective medium of information. In addition, the odds of negative attitudes towards people living with HIV were highest among those women with no education. The higher odds were nearly halved between NFHS 4 and NFHS 5, which might be due to the increase in the proportion of formal education among women and increased information, education, and communication activities on HIV/AIDS under NACO. However, there has been an increase in the odds of having a negative attitude toward people living with HIV among women in rural areas. The study done in 2017 [11] suggested that educational and socioeconomic setbacks should be overcome to impose a better and justified attitude toward HIV/AIDS. Improved health conditions, proper counseling, and knowledge are essential to break communication barriers and ignorance toward HIV/AIDS.

As per the conclusions of the study done in 2021 [20], there was a higher awareness among adolescent boys than in adolescent girls. Specific predictors of high awareness were also noted in the study, including higher age, higher education, and exposure to mass media. This shows that the males are beneficiaries of privileges, because of which there was better awareness. These findings are concordant with our models. The odds of believing in myths related to HIV/AIDS were highest among women with no education and those residing in rural areas. This can be due to the lesser opportunities and exposure to sources of information, such as mass media, etc, in rural areas.

With respect to the knowledge of prevention of HIV/AIDS, during NFHS 5, there were decreased odds among those women who had no education or had only primary education when compared to those with the same educational attainment during NFHS 4. This finding can result from various governmental and nongovernmental institutions' Information Education and Communication (IEC) Campaigns. However, the protective effect (adjusted odds ratio less than 1) of residing in the North and East zone had decreased and progressed more towards adjusted odds ratio one during NFHS 5 compared to that during NFHS 4. This finding can indicate the increase in disparity and the need to focus on spreading knowledge in these areas. In addition, there was a decrease in the odds of negative attitudes toward PLHIV among women with no education during NFHS 5 compared to that during NFHS 4.

Conversely, there was an increase in the odds of negative attitudes toward PLHIV among women residing in the North and East zone. Causes similar to those for comparative findings for knowledge of prevention of HIV/AIDS can explain the findings for odds for negative attitude towards PLHIV. Finally, there was an increase in the odds of beliefs in myths related to HIV/AIDS among those women residing in the East zone during NFHS 5 compared to that during NFHS 4. We made these comparisons based on the fact that the confidence intervals of the adjusted odds ratio do not overlap for these findings. Another method to assess the change can be by using formal time-series model to differentiate random fluctuations from actual systematic changes. However, the mentioned analysis is beyond the scope of this research.

Strengths of the study

This study had a population representative of the country. We were able to make a good model to show the change among determinants affecting the perception of the epidemiology of HIV/AIDS since 2015.

Limitations

Responses of the women to whom questions about HIV/AIDS were not asked could have made a statistically significant difference to our results. This is an analysis of secondary data; we could not assess other aspects of women's perceptions of the epidemiology of HIV/AIDS.

Recommendations for further studies

Further studies can be conducted at the grassroots level, with both quantitative and qualitative components, to obtain information and misinformation related to the epidemiology of HIV/AIDS among women. Comprehensive sex education among adolescents in rural areas can have a good positive impact.

## Conclusions

This study highlights the significance of various factors in shaping knowledge, attitudes, and beliefs related to HIV/AIDS. The findings underscore the importance of addressing regional disparities, particularly in rural areas, where individuals face higher likelihoods of insufficient knowledge, negative attitudes towards PLHIV, and belief in HIV/AIDS myths. Furthermore, the study identifies lower age groups and individuals with no education as vulnerable populations, emphasizing the need for targeted interventions to improve awareness and dispel misconceptions among these groups. Additionally, the study emphasizes the role of mass media in promoting accurate information and creating a protective factor for the correct perception of the epidemiology of HIV/AIDS. These findings contribute to a deeper understanding of the challenges and opportunities in HIV/AIDS prevention and education, highlighting the importance of comprehensive and tailored approaches to address the knowledge gaps and negative attitudes associated with HIV/AIDS.
